# Prevalence of smoking (cigarette and waterpipe) and its association with obesity/overweight in UAE and Palestine

**DOI:** 10.3389/fpubh.2022.963760

**Published:** 2022-10-21

**Authors:** Haleama Al Sabbah, Enas A. Assaf, Elias Dabeet

**Affiliations:** ^1^Department of Health Sciences, College of Natural and Health Sciences, Zayed University, Dubai, United Arab Emirates; ^2^Community Health Department, Faculty of Nursing, Applied Science Private University, Amman, Jordan; ^3^Science Department, Eastern Iowa College, Davenport, IA, United States

**Keywords:** smoking, waterpipe, obesity, university students, UAE, Palestine

## Abstract

**Aim:**

This study aimed to assess the prevalence rate of smoking behavior (cigarette and waterpipe) and its association with obesity/ overweight among university students in the United Arab Emirates (UAE) and Palestine.

**Methods:**

A cross-sectional study was conducted at a convenient sample of 10 largest universities in the West Bank- Palestine and Dubai- UAE. In total, 3800 students were randomly selected from the universities, with an 87.6% response rate. A self-administered questionnaire was used to collect the data. The key measures were: waterpipe smoking, weight, height, cigarette smoking, dieting to reduce weight, and perception and knowledge related to tobacco waterpipe smoking (TWP). Body Mass Index (BMI) was calculated using the WHO cutoffs.

**Results:**

The analysis included 3,327 students (54% from the West Bank and 46% from Dubai). About 16% of students in Dubai and 18% of students in the West Bank smoke cigarettes. Quarter (26%) of the students in Dubai and 32% of the students in West Bank smoke water pipes. 17% of students in Dubai and 18% of students in the West Bank reported that they smoke waterpipes to reduce their weight. Waterpipe smoking was found to have a significant positive association with obesity/overweight (*p* < 0.001).

**Conclusions:**

Smoking is very common among university students. Waterpipe smoking was associated with obesity. More research is still needed in this field to better understand the relationship between cigarette smoking and obesity.

## Introduction

Tobacco use is the leading preventable cause of morbidity and mortality (1). Tobacco use is responsible for more than 8 million deaths annually worldwide (2). According to reports from the WHO, more than 80% of smokers are found in low-to middle-income countries (2). Tobacco waterpipe smoking (TWP) (hookah, shisha), is perceived as less toxic than cigarettes (3) and is rising dramatically among young people in the Middle East (3, 4). The practice of TWP smoking has strong roots in the Arab world. It is practiced socially in commercial cafes. It is popular among Arab women, as it is less stigmatized than cigarette smoking and enjoys greater social acceptability (4, 5). A widespread myth about TWP smoking is that the passage of smoke through the water in TWPs “purifies” the smoke of harmful constituents (6).

The most severe well-known health outcomes associated with smoking are cancer, heart disease, stroke, lung disease (1), nicotine addiction (7), anxiety disorders, and depression (8). Exposure to nicotine after TWP smoking is at almost the same levels like those associated with cigarette smoking (9). In addition, long-term use of TWPs with regular cigarette smoking is associated with several health problems including pulmonary, gastrointestinal, bladder, cardiovascular, and hematological diseases (10). TWP smoking was positively associated with obesity and increased body waist circumference (10, 11). Other dangers include infections such as tuberculosis, hepatitis C, pulmonary aspergillosis (11, 12), Helicobacter pylori infection since it's transmitted by saliva (13), Middle East Respiratory Syndrome (MERS-CoV) (14), and COVID-19 (15), which have been assumed to spread from pipe sharing.

Although the hazards of TWP smoking have been well characterized, they are not yet understood by the general population, even in the Arab world. Studies conducted among the general adult population, high school students, university students, and even pregnant women in the Middle East unanimously reported that TWP smoking is generally perceived as much less detrimental than cigarette smoking (3, 4, 9, 14, 15).

Factors that have been strongly linked to the initiation of TWP smoking include social and academic stress, pleasure-seeking, boredom, peer pressure, and the intimacy between persons of the opposite sex generated by TWP sharing (16). Globally, stress, peer pressure, and boredom are common phenomena in students, placing the group at potential risk of taking up TWP smoking (17, 18). An understanding of the awareness, perceptions, practice, and dependence of this group as regards TWP smoking is therefore of paramount importance, and the primary objective of this study. In addition to sociodemographic characteristics, income and lifestyle are significant influences on smoking (19). Although TWP smoking is very popular in Dubai cafes and restaurants, some studies were done on the prevalence and its determinants among university students who smoke TWP. A study investigating the factors influencing TWP smoking among dental students in Sharjah found that peer pressure, sibling smoking, and the social acceptability of TWP smoking were the key determinants. And the most common factor behind the initiation of TWP was mainly stress (20). Another study was conducted on Sharjah university students to find that 16.3% of students were addictive to TWP (21). Additionally, a recent study on nicotine vaping revealed that 15.1% of smokers use solely vapes and do not use any other sources of nicotine (22). One study conducted in Palestine to assess the prevalence of TWP smoking among university students found that 24.2% did so, with the rate among men significantly higher than that of women (23).

The socioeconomic status and lifestyles of university students in Dubai and the West Bank and the effect of TWP smoking on body weight and obesity were never investigated. The prevalence of smoking reflects the magnitude of the problem. Determining it is important since it provides a basis for the planning of public health actions and preventive measures. This study filled the gaps in terms of sociodemographic differences as it was conducted at 5 universities on the West Bank and 5 universities in Dubai. In this study, we analyzed the prevalence of TWP smoking among university students and its association with obesity and compared the results between students with high socioeconomic status and high quality of life living in the United Arab Emirates with students living under occupation with lower socioeconomic status and increased stress in Palestine.

## Methods

### Study design and settings

A cross-sectional study was conducted at a convenient sample of largest universities in Dubai and West Bank (5 in Dubai-UAE and 5 in the West Bank-Palestine). The questionnaire was adapted from one used previously in similar populations and translated into Arabic. A pilot study on 20 students was conducted in each region to ensure that all questions are clear. According to the results of the pilot study and students' feedback, the questionnaire was revised before data collection. Some questions and some words were changed to make them more understandable (e.g., Nargila was changed to Shisha). Prior to data collection, the study was approved by Zayed University Research Ethics Committee (Ref. Code: ZU14_051_F) and consent was obtained from the participants. Permission was obtained from each target university before the data collection. Data collection was completed during the Spring semester of 2015.

### Population and sampling

The sampling method used was proportional, which means that the number of students chosen from each university or gender is proportional to the number of students in that university or gender. The proposed sample size was 3,800 students (1,900 from the West Bank universities and a similar number from Dubai universities), with a margin of error of 5 and 95% confidence level to ensure sufficient power for bivariate and multivariate analysis. The study sample consisted of students from five universities in the West Bank (Al Quds University, An Najah University, Birzeit University, Arab American University, and Bethlehem University) and five universities in Dubai (Zayed University, Canadian University, Dubai University, American University of Dubai, and Ghurair University).

### Data collection process and instruments

Students were approached on campus by trained data collectors who explained the study objectives and obtained the student's consent. The students' anonymity was maintained throughout the process. A self-reported questionnaire (20 min to be filled out) was handed to the students. In total, 3,327 questionnaires were completed, with an overall 87.6% response rate. Anthropometric measurements for Weight, Height and Waist circumference were obtained by the trained data collectors from a sub-sample of the participating students who completed the questionnaire. The measurements were taken in private rooms at each university (50 students per university) from a randomly selected sample of 500 students (250 from the West Bank and 250 from Dubai). However, 211 from Dubai and 262 from the West Bank agreed to participate in the measurements for the sub-sample. Measurements for weight, height, and waist circumference from a sub-sample of 500 students were taken to verify self-reported weight and height and to determine the relationship between abdominal obesity and waterpipe smoking by measuring the waist circumference of these students (waterpipe smokers and non-smokers).

A self-reported questionnaire composed of several parts including socio-demographic characteristics, The Fagerstrom Test for Nicotine Dependence (FTND) for cigarettes (24), TWP smoking history, practices including frequency and time spent, attitudes and awareness of the TWP hazards, Lebanese waterpipe dependent scale (LWDS-11) test for TWP dependence. Cronbach's alpha coefficients range from 0.55 to 0.88 for subscales; equal to 0.83 which is considered to be acceptable. Test-retest coefficients (Spearman correlation coefficients) were r = 0.92 (*p* < 10^−4^). The scale is written in Arabic (25). Body Mass Index was calculated by dividing weight in kg by height in meters squared (kg/m^2^). BMI categorized into Underweight (BMI < 18.5), Normal (BMI = 18.5–24.9), Overweight (BMI = 25–29.9), and Obese (BMI ≥30, following the WHO criteria (26). Waist circumference (WC) measurement was used to assess abdominal obesity and central obesity based on WHO cutoff criteria (WC = 88 for females and ≥ 102 for males) (27).

### Data analysis

The data were entered and analyzed using SPSS version 22.0. In descriptive analysis frequency and percentages were presented. Chi-square test and G-test were used to test the associations between independent and dependent variables (BMI and smoking). A *p*-value of < 0.05 is considered significant.

## Results

### Socio-demographic characteristics

Overall, 3,327 university students participated in the study (54% in the West Bank, 46% in Dubai) with a response rate of 87.6%. More than half of the participants (58.5%) were 20–22 years old (61% in West Bank and 47% in Dubai). More females (65.1%: 66.6% in West Bank and 59.1% in Dubai) participated in this study than males. The majority (85%) are single (Table 1).

**Table 1:** Socio-demographic and weight status characteristics.

**Characteristic**		**Dubai**		**West Bank**		**Overall**	
		***N***	**%**	***N***	**%**	***N***	**%**
Age	18–19	437	28.4	579	32.4	1016	30.5
	20–22	729	47.4	1096	61.3	1825	54.9
	23–25	235	15.3	101	5.6	336	10.1
	> 25	137	8.9	13	0.7	150	4.50
	**Total**	**1538**	**100**	**1789**	**100**	**3327**	**100**
Gender	Male	645	40.9	618	33.4	1263	36.9
	Female	932	59.1	1230	66.6	2162	63.1
	**Total**	**1577**	**100**	**1848**	**100**	**3425**	**100**
Martial status	Single	1336	84.7	1591	85.8	2927	85.3
	Married	152	9.6	105	5.7	257	7.5
	Engaged	77	4.9	148	8.0	225	6.6
	Divorced	13	0.8	9	0.5	22	0.6
	**Total**	**1578**	**100**	**1853**	**100**	**3431**	**100**
Colleges	Foundation	149	9.6	6	0.3	155	4.5
	Economy & business	506	32.6	536	28.9	1042	30.5
	Social science	117	7.5	441	23.7	558	16.4
	Science	148	9.5	497	26.8	645	18.9
	Other	634	40.8	377	20.3	1011	29.7
	**Total**	**1554**	**100**	**1857**	**100**	**3411**	**100**
Waterpipe smoking history (years)	< 2 years	42	12.9	50	11.7	92	12.2
	2–4 years	150	46.0	257	60.2	407	54.1
	5–7 years	85	26.1	85	19.9	170	22.5
	> 7 years	49	15.0	35	8.2	84	11.2
	Total	326	100.0	427	100	753	100

### Prevalence of smoking (cigarette and TWP), playing sports, and dieting

Table 2 shows that 15.7% (29.6% of males and 6% of females) from Dubai and 17.5% (46% of males and 3.3% of females) from the West Bank students smoke cigarettes (*p* < 0.001). Moreover, 5.6% (9.9% of males and 2.6% of females) from Dubai while 8.7% (16.8% of males and 4.6% of females) from the West Bank constantly/ daily smoke waterpipe (*p* < 0.001). Sport practicing was more prevalent in Dubai than in the West Bank, the difference was significant (*p* < 0.001). About 26.8% (41.4% of males and 16.8% of females) from Dubai while 16% (25.3% of males and 11.3% of females) from the West Bank were practicing sports regularly (*p* < 0.001). Dieting to reduce weight among students living in Dubai was about twice that of those living in the West Bank, 21.1 % (20.5% of females and 21.6% of males) and 13.9% (12.4% of females and 14.7% of males) respectively, (*p* < 0.001) (Table 2).

**Table 2:** Prevalence of smoking (cigarette and TWP), playing sports, and dieting.

	**Dubai**						**West Bank**						***P*-value**
	**Males**		**Females**		**Total**		**Males**		**Females**		**Total**		
	***N***	**%**	***N***	**%**	***N***	**%**	***N***	**%**	***N***	**%**	***N***	**%**	
**Smoke cigarettes**													<0.001
Yes	191	29.6	56	6	247	15.7	284	46	40	3.3	324	17.5	
No	454	70.4	876	94	1330	84.3	334	54	1190	96.7	1524	82.5	
**Total**	645	100	932	100	1577	100	618	100	1230	100	1848	100	
**Smoke waterpipe**													<0.001
Yes, constantly	64	9.9	24	2.6	88	5.6	104	16.8	57	4.6	161	8.7	
Yes, sometimes	186	28.8	131	14.1	317	20.1	226	36.6	192	15.6	418	22.6	
No	395	61.2	777	83.4	1172	74.3	288	46.6	981	79.8	1269	68.7	
**Total**	645	100	932	100	1577	100	615	100	1230	100	1848	100	
**Play sports**													<0.001
Regularly	265	41.4	155	16.8	420	26.8	156	25.3	138	11.3	294	16	
Irregularly	324	50.6	582	63.1	906	58	359	58.2	796	65.1	1155	62.8	
I don't practice	51	8	186	20.2	237	15.2	102	16.5	289	23.6	391	21.3	
**Total**	640	100	923	100	1563	100	617	100	1223	100	1840	100	
**Diet to reduce weight**													<0.001
Yes	131	20.5	200	21.6	331	21.1	76	12.4	181	14.7	257	13.9	
No, my weight is fine	303	47.3	375	40.4	678	43.2	357	58	667	54.3	1024	55.5	
No, but I want to reduce weight	135	21.1	265	28.6	400	25.5	88	14.3	272	22.1	360	19.5	
No, I want to gain weight	71	11.1	88	9.5	159	10.1	94	15.3	109	8.9	203	11	
**Total**	640	100	928	100	1568	100	615	100	1229	100	1844	100	

### Prevalence of obesity and abdominal obesity

Most of the reported BMI was within the normal range. While obesity was more prevalent in Dubai. The prevalence of self-reported obesity/overweight among students living in Dubai was almost doubled of those living in the West Bank (9.4 /22.2% vs. 2.9 /14.3% respectively). Whereas the prevalence of measured obesity/overweight among students living in Dubai and students living in the West Banks was 8.5/21.8 and 3.8/14.1%, respectively (Table 3). The self- reported obesity/overweight was slightly more than the obesity/overweight measured in the sub-sample; the difference was not significantly different (*p* > 0.05). Moreover, Table 3 shows the sex-specific waist circumference according to the WHO cutoffs and the increased risk of metabolic complications. Results from the measured waist circumference of the sub-sample found that abdominal obesity (substantially increased risk of metabolic complications) among male and female students living in Dubai was 10 and 11%, respectively. Whereas the abdominal obesity (substantially increased risk of metabolic complications) among females (9.1%) living in the West Bank was almost doubled of the males (5.4%) (Table 3).

**Table 3:** Self-reported BMI, measured BMI and measured waist circumference.

**Characteristic**		**Dubai**		**West Bank**		**Overall**	
		***N***	**%**	***N***	**%**	***N***	**%**
Self-reported Weight status	Underweight	151	9.6	162	8.8	313	9.2
	Normal Range	921	58.8	1361	74.0	2282	67.0
	Overweight	347	22.2	263	14.3	610	17.9
	Obese	146	9.4	54	2.9	200	5.9
	**Total**	**1565**	**100**	**1840**	**100**	**3405**	**100**
Measured Weight status	Underweight	23	10.9	22	8.4	45	9.5
	Normal Range	124	58.8	193	73.7	317	67.1
	Overweight	46	21.8	37	14.1	83	17.5
	Obese	18	8.5	10	3.8	28	5.9
**Measured Waist Circumference (WC)**	**Men**	**Women**	**Men**	**Women**	**Men**	**Women**	
	***N*** **(%)**	***N*** **(%)**	***N*** **(%)**	***N*** **(%)**	***N*** **(%)**	***N*** **(%)**	
**Increased:** >94-102 cm (M); >80-88 cm (W)	12 (12.0)	18 (15.3)	19 (17.1)	39 (25.3)	32 (15.1)	57 (20.4)	
**Substantially increased (abdominal obesity:** >102 cm (M); >88 cm (W)	**19 (19.0)**	**13 (11.0)**	**6 (5.4)**	**14 (9.1)**	**17 (8.1)**	**29 (10.4)**	
**Not increased:** ≤94 cm (M); ≤ 80 cm (W|)	69 (69)	87 (73.7)	86 (77.5)	101(65.6)	162 (76.7)	193 (69.2)	
**Total**	**100 (100)**	**118 (100)**	**111 (100)**	**154 (100)**	**211 (100)**	**279 (100)**	

M, men; W, women.

### Perceptions and attitude toward waterpipe smoking

According to the findings, 55% of Dubai students and 61% of West Bank students believe that smoking a waterpipe is more acceptable than smoking cigarettes. About 18% of Dubai students and 13% of West Bank students stated that smoking waterpipe is less harmful than smoking cigarettes. In addition, about 18% of Dubai students and 12% of West Bank students believe that smoking waterpipe causes less damage to the respiratory system than cigarettes. About 27% of Dubai students and 23% of West Bank students have a perception that waterpipe contains less nicotine than cigarettes. As to whether smoking is a good habit or not, about 8% of Dubai students and 11% of West Bank students believe that it is a good habit. The findings also show that 14% of the students in Dubai and 18% of students from the West Bank believe that waterpipe is more acceptable for females than smoking cigarettes. On the other hand, about 48% of students from Dubai and 47% of students from the West Bank believe that it's not permissible for females to smoke waterpipes and cigarettes. Even if they are sick and in bed, 14 percent of Dubai students and 21% of West Bank students said they will still smoke waterpipes. About 14% of Dubai students and 22% of West Bank students stated that they would be willing to forego food in exchange for waterpipe smoking. To lose weight, 17% of Dubai students and 18% of West Bank students smoke waterpipes. Because it has good taste, 87% of Dubai students and 91% of West Bank students smoke waterpipes (Table 4).

**Table 4:** Student's perception toward TWP smoking in Dubai and West Bank.

	**Dubai**		**West Bank**		**Overall**	
	***N***	**%**	***N***	**%**	***N***	**%**
Shisha is a good habit	123	7.8	205	11.1	236	10.4
Shisha is less harmful than cigarettes	289	18.4	244	13.2	326	14.4
Shisha causes less damage to the respiratory system than cigarettes	228	17.5	228	12.4	299	13.2
Shisha contains less nicotine than cigarettes	420	27.1	410	22.5	509	22.8
Shisha is more acceptable more than cigarettes	848	55.0	1086	60.6	1321	59.9
Female may smoke shisha but it is not permissible for her to smoke cigarettes	211	13.7	329	18.0	388	17.3
Female may not smoke shisha and is not permissible for smoking cigarettes	747	48.1	851	46.5	1024	45.6
Ready for not eating food in exchange for smoking shisha	48	14.4	99	22.5	147	18.9
To Reduce Weight	56	17.2	78	17.7	134	17.5
It tastes nice	282	87.3	394	90.7	676	89.2

### Associations between weight status and smoking (cigarettes and TWP)

This study found that there is an association between reported BMI and smoking (cigarettes and TWP) among university students in both Dubai and West Bank (*p* < 0.001). Furthermore, there were significant associations between measured BMI and smoking cigarettes among students at Dubai universities. In contrast, there is no significant association between measured BMI and smoking cigarettes among West Bank university students. There is an association between measured BMI and TWP smoking among students at Dubai universities. There was no association between measured BMI and smoking TWP among students at the West Bank universities. In both Dubai and the West Bank, there is an association between measured waist circumference (cm) and smoking cigarettes. When it came to TWP smoking, it was found that there was an association between waist circumference (cm) and smoking among Dubai students, but not among West Bank students (Table 5).

**Table 5:** The associations between weight status and smoking (cigarettes and TWP).

**Dependent variable**	**Independent variable**	**TEST**	**Test value**	***P-value***
**Dubai results**				
Reported BMI	Smoking Cigarettes	*χ^2^*	18.38	<0.001^*^
	Smoking TWP	*χ^2^*	23.03	0.001^*^
Measured BMI	Smoking Cigarettes	*G*	6.64	0.084
	Smoking TWP	*G*	20.08	0.003^*^
Measured Waist Circumference	Smoking Cigarettes	*G*	22.31	<0.001^*^
	Smoking TWP	*G*	32.34	<0.001^*^
**West-bank results**				
Reported BMI	Smoking Cigarettes	*χ^2^*	13.32	0.004^*^
	Smoking TWP	*χ^2^*	41.03	<0.001^*^
Measured BMI	Smoking Cigarettes	*χ^2^*	1.90	0.387
	Smoking TWP	*G*	4.54	0.338
Measure Waist Circumference	Smoking Cigarettes	*G*	50.90	<0.001^*^
	Smoking TWP	*G*	6.38	0.605
**Overall results**				
Reported BMI	Smoking Cigarettes	*χ^2^*	29.95	<0.001^*^
	Smoking TWP	*χ^2^*	38.46	<0.001^*^
Measured BMI	Smoking Cigarettes	*G*	4.52	0.104
	Smoking TWP	*G*	9.69	0.046^*^
Measure Waist Circumference	Smoking Cigarettes	*G*	54.39	<0.001^*^
	Smoking TWP	*G*	28.87	<0.001^*^

G-test (Likelihood ratio test) used if the assumptions of Chi-square test dis not achieved. (If there is an expected value frequency < 5.

* significant at 5%.

G, likelihood ratio test used if chi-square test assumptions not achieved.

Weight, with “kg” Unit.

BMI, with “kg/m^2^” Unit.

## Discussion

Smoking (cigarette and TWP) was found to be influenced by income, lifestyle, and sociodemographic variables (16, 28). This study aims to compare two different sociodemographic factors in terms of income and lifestyle among university students who smoke TWP and have a high BMI (West Bank—Palestine and Dubai—United Arab Emirates). The study findings showed that when it came to TWP smoking and BMI, 26% were overweight and 12% were obese, and there is a significant association between TWP smoking and obesity. This was consistent with studies conducted in Qatar, Syria, and Pakistan, respectively (10, 29, 30). This could be related to the fact that TWP smoking is usually associated with a long period of inactivity, as well as social contact in cafes and restaurants, which leads to increased food consumption and long periods of inactivity (31). On the other hand our study found that the students believe that smoking can reduce weight.

According to a study conducted in Lebanon, TWP smoking is connected with poor eating habits among adolescents, as most participants tend to eat unhealthy foods such as high-fat foods while also smoking TWP, which can lead to weight gain (32). According to one early cross-sectional study, TWP smokers may have metabolic syndromes when compared to non-smokers, as TWP smokers have higher levels of triglyceride, blood pressure, glucose, and abdominal obesity than non-smokers (10). Another study on the effects of TWP smoke on animals (rats) found that mice exposed to TWP smoke had higher body weight, belly circumference, fasting hyperglycemia, and systolic blood pressure than non-exposed (control) mice (33).

Using measured BMI, our study found a significant difference between Dubai and West Bank students in terms of obesity and overweight; Dubai students complain of overweight and obesity at higher rates than West Bank students. Our research, on the other hand, found that TWP smokers in the West Bank were greater than those among Dubai students. This could be related to the stark variations in lifestyle between the two locations. Most students in the West Bank rely on public transportation, and an active lifestyle to move to the university or even inside the university campus. They also have limited daily pocket money, therefore they prefer to have restricted spending even when smoking TWP in cafés or restaurants. As a result, an unhealthy diet is not a daily habit for them; it is seen as fancy cuisine (34).

In the West Bank, people have men's cafés where they serve TWP and drinks at a low cost. As a result, students can socialize with each other with little expense. Whereas in Dubai, students use private transportation to get to university, rarely walk between collages within universities, and with a higher socioeconomic status, students tend to have higher standard living costs, and their expenditures on restaurants and unhealthy diets are significantly higher than those of West Bank students. This is consistent with a recent Turkish study comparing university students at public and private institutions and TWP smoking, to find out that students having a car, a large pocketbook, and studying in a private university would be more likely to smoke TWP in upscale restaurants and cafés (35).

When comparing the results of our study on athletic practices, however, there were substantial variations between students from the West Bank and Dubai. In both males and females, more students in Dubai participated in sports on a regular and irregular basis than in the West Bank. This would suggest that our first explanation for the BMI disparities is based on lifestyle and daily activities, which are influenced significantly by socioeconomic class between the two places, similar to a study conducted in Turkey (35). Another explanation is that the West Bank's movement stress as a result of political occupation and suppression limits sports activity and commitment to sports centers and clubs after university study attendance, particularly for students who live in villages and rural areas because sports centers are primarily located in large cities (36). When looking at the results regarding the need to go into a special diet to lose weight, interestingly the results were consistent with the other findings of this study, as there was a significant difference between students in both areas, with those from Dubai being more concerned and feeling the need to lose weight than those from the West Bank, which is consistent with the increase in BMI between the two areas. Furthermore, there was an association between TWP smoking and waist circumference among Dubai students. In one meta-analysis study (37), smoking was found to be associated with an increase in BMI and waist circumference. In one study conducted in Pakistan, TWP smoking was found to be significantly associated with metabolic syndrome and abdominal obesity (10).

Our study found several interesting opinions concerning TWP smoking, such as the average student thought that TWP smoking is more acceptable than smoking a cigarette, and a quarter of them thought that shisha contains less nicotine than a cigarette. This was similar to what was found in a systemic review study assessing the motives for TWP smoking (38). While over a third of the students stated that they would smoke water pipe even if they were un-well and in bed. In addition to their willingness to forego food in exchange for smoking a water pipe, those two attitudes could be signs of nicotine addiction. According to findings from a study in Lebanon (39), TWP smoking can cause nicotine addiction due to the frequency of smoking, the length of smoking sessions, and the number of TWPs smoked.

Female smoking attitudes were also found to differ between the two groups, the statement “female may smoke shisha but it is not permissible for her to smoke cigarettes” received more acceptance on the West Bank than in Dubai. This could be related to the fact that in most regions of the West Bank, TWP smoking is culturally acceptable for females in cafés or outside their homes, as discussed in a previous study among university students (23).

On the other hand, in Dubai, where there is a mixed cultural society with various nationalities and modernized circumstances, female TWP smoking may be more acceptable. Although it was not specifically mentioned, a recent study in the UAE (40) found a significant increase in the incidence and popularity of TWP smoking among youth and young female adults. TWP was more popular among Dubai students since it is less harmful than cigarettes, contains less nicotine, and has a lower respiratory effect. This was supported by other studies (41, 42). This would predict an increase in TWP smoking epidemiology among young adults in Dubai. In addition, it helps in initiating health promotion policies in Dubai regarding the effect of TWP smoking on overall health. It might be important to raise some of the study's limitations, one of which is the social desirability bias among university students, who are less likely to admit their smoking habits, particularly females. Furthermore, students may respond to attitude-related questions in a variety of ways.

## Conclusions

Socioeconomic differences were markedly found to influence BMI and TWP smoking between West Bank -Palestine and Dubai-United Arab Emirates students. Despite this, the prevalence remains high in both regions. Programs aimed at education, prevention, and intervention for waterpipe use are needed to address the high prevalence rate of water pipe smoking and this growing public health concern. More research is still needed in this field to highlight the relationship between cigarette smoking and obesity.

## Data availability statement

The original contributions presented in the study are included in the article/supplementary material, further inquiries can be directed to the corresponding author.

## Ethics statement

The studies involving human participants were reviewed and approved by Zayed University (ZU) Ethical Committee (Ref. Code: ZU14_051_F). The patients/participants provided their written informed consent to participate in this study.

## Author contributions

HA designed and wrote the first draft of the manuscript and was the PI for the research grant. EA contributed in updating the draft and references. HA and ED conducted the study in both Dubai-UAE and West Bank-Palestine and performed the data analysis. All authors contributed to the article and approved the submitted version.

## Funding

The present study was supported by Zayed University RIF grant (grant no. R14121).

## Conflict of interest

The authors declare that the research was conducted in the absence of any commercial or financial relationships that could be construed as a potential conflict of interest.

## Publisher's note

All claims expressed in this article are solely those of the authors and do not necessarily represent those of their affiliated organizations, or those of the publisher, the editors and the reviewers. Any product that may be evaluated in this article, or claim that may be made by its manufacturer, is not guaranteed or endorsed by the publisher.
